# The integration of probabilistic information during sensorimotor estimation is unimpaired in children with Cerebral Palsy

**DOI:** 10.1371/journal.pone.0188741

**Published:** 2017-11-29

**Authors:** Claire Chambers, Taegh Sokhey, Deborah Gaebler-Spira, Konrad P. Kording

**Affiliations:** 1 Sensory Motor Performance Program, Shirley Ryan Abilitylab, Chicago, Illinois, United States of America; 2 Biomedical Engineering, Robert R. McCormick School of Engineering and Applied Sciences, Northwestern University, Chicago, Illinois, United States of America; 3 Department of Physical Medicine and Rehabilitation, Feinberg School of Medicine, Northwestern University, Chicago, Illinois, United States of America; 4 Department of Biological Sciences, Northwestern University, Evanston, Illinois, United States of America; Boston Children’s Hospital / Harvard Medical School, UNITED STATES

## Abstract

**Background:**

It is important to understand the motor deficits of children with Cerebral Palsy (CP). Our understanding of this motor disorder can be enriched by computational models of motor control. One crucial stage in generating movement involves combining uncertain information from different sources, and deficits in this process could contribute to reduced motor function in children with CP. Healthy adults can integrate previously-learned information (prior) with incoming sensory information (likelihood) in a close-to-optimal way when estimating object location, consistent with the use of Bayesian statistics. However, there are few studies investigating how children with CP perform sensorimotor integration. We compare sensorimotor estimation in children with CP and age-matched controls using a model-based analysis to understand the process.

**Methods and findings:**

We examined Bayesian sensorimotor integration in children with CP, aged between 5 and 12 years old, with Gross Motor Function Classification System (GMFCS) levels 1–3 and compared their estimation behavior with age-matched typically-developing (TD) children. We used a simple sensorimotor estimation task which requires participants to combine probabilistic information from different sources: a likelihood distribution (current sensory information) with a prior distribution (learned target information). In order to examine sensorimotor integration, we quantified how participants weighed statistical information from the two sources (prior and likelihood) and compared this to the statistical optimal weighting. We found that the weighing of statistical information in children with CP was as statistically efficient as that of TD children.

**Conclusions:**

We conclude that Bayesian sensorimotor integration is not impaired in children with CP and therefore, does not contribute to their motor deficits. Future research has the potential to enrich our understanding of motor disorders by investigating the stages of motor processing set out by computational models. Therapeutic interventions should exploit the ability of children with CP to use statistical information.

## Introduction

Cerebral Palsy (CP) describes a group of permanent disorders of the development of movement and posture, causing activity limitation, that are attributed to non-progressive disturbances that occurred in the developing fetal or infant brain [1,2]. CP is a lifelong condition without a cure or treatments which can fully alleviate symptoms. It affects an estimated 1.5 to 4 per 1000 children [3–6] and is among the most common causes of disability in children [7,8]. However, despite the degree of motivation to develop better treatments, we currently do not have a complete or, in particular, a computational understanding of the condition.

CP has been studied by classifying its symptoms and by addressing the neural correlates of symptoms. Symptoms can be divided into positive symptoms, characterized by increased muscle tone and movement, including spasticity, rigidity, dystonia, chorea, and tremor; and negative symptoms, characterized by a lack of muscle tone and movement, including weakness, poorly selective motor control, ataxia, and dyspraxia [2,9–11]. Broadly speaking, symptoms may be caused by damage to the pyramidal tract, which are nerve fibers originating in motor cortex that extend to the brain stem, this subtype being associated with spasticity [12]. Symptoms may also be caused by damage to regions outside the pyramidal tract including basal ganglia and thalamus, with this form of damage being associated with dyskinetic movement and ataxia [13]. Brain imaging in CP patients has revealed abnormal activity in cortical regions which support somatosensation, motor, and goal-directed behaviors [14]. Abnormal somatosensory activity in early electrophysiological responses [15] and reduced spatial extent and magnitude of cortical activity [14] have been observed in CP patients. In hemiplegic patients, differences in somatosensory oscillatory activity have been observed between affected and non-affected hemispheres [16]. Measures of connectivity are also affected in CP patients, with findings of reduced long-range inter-region connectivity [17], and reduced thalamocortical and corticospinal connectivity [18–21]. Findings suggest an inverse correlation between connectivity measures and symptom severity and an increase in connectivity in response to therapy [20,21]. Description of the symptoms and the neurological markers of CP have contributed to our understanding of the disease.

Independently of brain injury, motor dysfunction can also be thought of in terms of the computations which underlie motor control. Models outline processing stages that allow healthy adults to produce a successful movement towards a sensory target [22,23]. For example, producing a movement can be described formally as containing a sensory processing stage (‘where is the target?’), which continually updates an internal model or representation of body position (‘where am I?’), and a stage which executes the motor command [24,25]. These models can be applied to motor disorders by examining the processing stages which are interrupted by illness and those which are preserved [26,27]. Principles derived from the literature on computational motor control have the potential to inform clinical practice through development of behavioral task-based treatments that target the stages of processing which are affected [28]. Further work is needed to bridge the gap between the computational understanding of how we execute movements and how this is disrupted in childhood motor disorders.

The role of sensory processing has been underlined in recent definitions of CP [2]. Children with CP have been found to have reduced visual and tactile sensitivity [29–31]. Impaired sensory feedback on the body’s position and motor action during movement is likely to contribute to reduced accuracy of movement in motor disorders [32,33]. In turn, poor motor control might cause children with CP to be deprived of successful learning experiences needed to develop motor skills in early life [34]. Therefore, impaired sensory processing may directly impact motor control and in turn prevent motor learning.

It should be noted that, in the current report, our discussion of sensory processing and sensory integration does not pertain to Sensory Integration Therapy, which prescribes interventions for tactile reactivity in the sensitive child [35]. Instead, we are interested in how children with CP bring together different sources of statistical information to inform their movements.

Some of the computations needed for successful motor function are preserved in CP. For example, children with CP retain the ability to learn and show improvements in movement accuracy with practice, although less than in typically-developing (TD) children [36]. Children with CP have intact internal models of their own movement variability, being able to take into account their own motor variability when moving to reach a target [37]. Delineating which functions are intact and impaired in CP will help to build better models of the disease.

One crucial element of successful motor control, which remains uninvestigated in CP patients, is the integration of information from different sources. The information that arrives at the senses contains uncertainty and cannot be fully relied on. Therefore, when deciding how to move, we are faced with the task of bringing together different sources of information [22]. When faced with this problem, healthy adults integrate current sensory information with prior knowledge in a manner which is well described by the rules of Bayesian inference [38,39]. This simply means that we weigh sources of information according to their relative uncertainty, with more certain information having a greater influence on sensorimotor estimation. Findings of Bayesian integration in motor behavior mean that healthy brains either explicitly or implicitly implement Bayesian computations [40,41].

It may be the case that Bayesian computations are disrupted in CP. However, there is some evidence that children with CP can use biofeedback in order to better regulate their movements [42,43]. While this suggests that children with CP can integrate information to control movement, these experiments do not explicitly quantify use of probabilistic information. Therefore, it is currently unknown whether Bayesian sensorimotor integration is disrupted in CP.

Here, we investigate whether CP involves a deficit in Bayesian integration. In our paradigm, we examine the use of probabilistic information to perform a simple sensorimotor estimation task, previously used in adults to examine sensorimotor integration under uncertainty [38,39,44]. Visual targets were drawn from a prior distribution and subjects were shown uncertain sensory information about each target, in a task where they inferred target location. We examined whether children with CP and TD controls differed in their use of the prior and the likelihood in their decisions.

## Methods

### Experimental details

**Fig 1:**
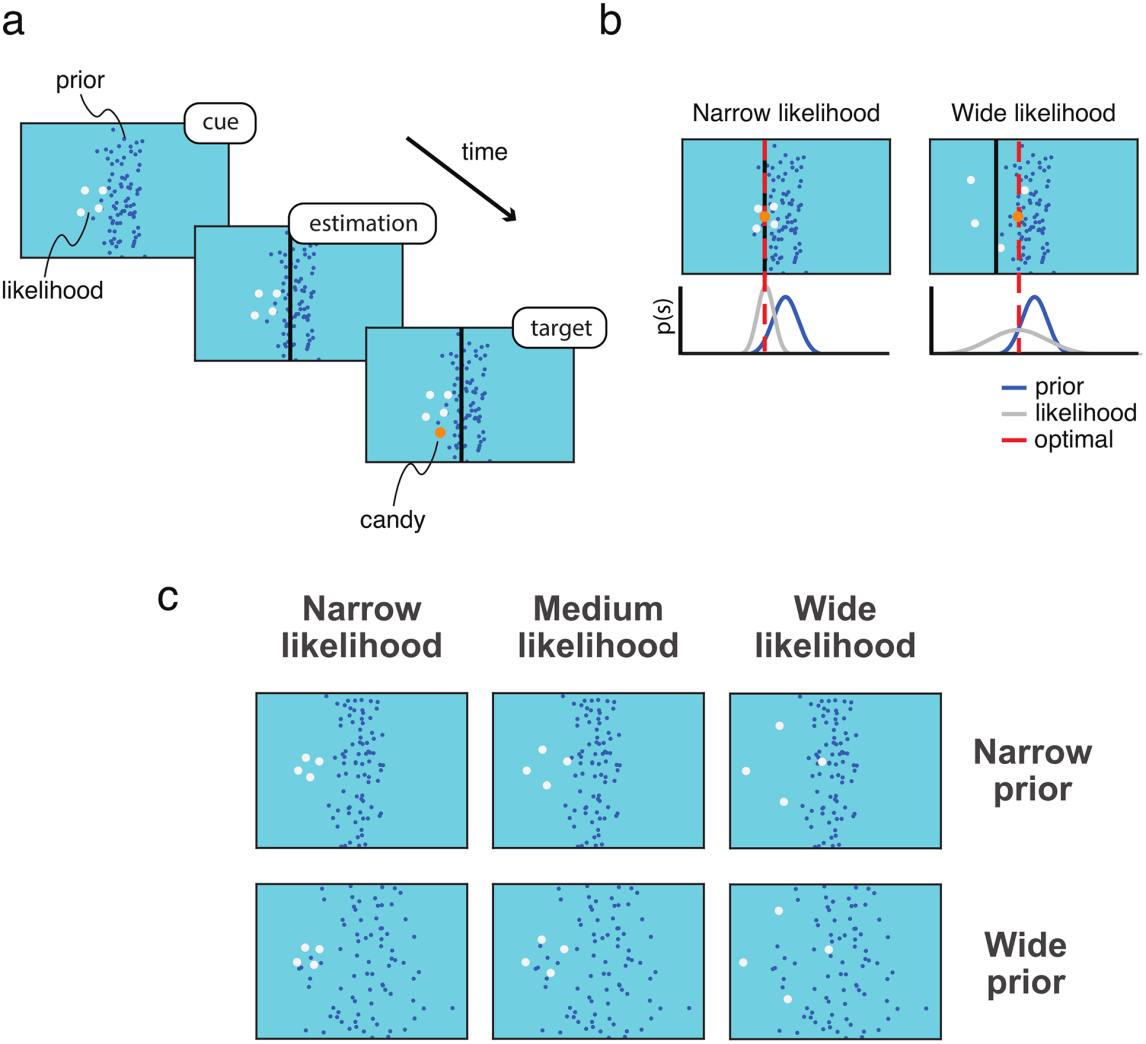
Experimental task. (**a**) Experimental protocol. Subjects were shown a visual cue (likelihood) with experimentally controlled uncertainty (splash), created by a hidden target (candy) drawn from a prior distribution. Subjects were told that the splash was created by candy falling into a pond. Subjects were prompted to place a net where the hidden target fell, and were then shown feedback on target location. (**b**) Relying on the likelihood. A simple strategy would be to rely entirely on likelihood information by pointing at its centroid on each trial. While this strategy is close to optimal when the likelihood is precise or narrow (left), this strategy is less successful when the likelihood is wider (right), as samples from the likelihood become a less reliable indicator of target location and the optimal estimate shifts closer to the prior mean. The optimal strategy involves weighing prior and likelihood information according to their relative uncertainties (**c**) Experimental design. In order to quantify integration of the prior and likelihood, we measured reliance on the likelihood (*Estimation slope*) under different conditions of prior variance and likelihood variance. The prior could be narrow or wide, and the likelihood narrow, medium, or wide.

Our task was designed to examine use of probabilistic information during sensorimotor estimation (Fig 1a) [38,44,45]. Previous findings indicate that during sensorimotor estimation, adults use sensory information in conjunction with prior information in a manner which resembles Bayesian integration, characterized by a weighing of information according to its relative uncertainty. In this study, we adapted a previously-used experimental protocol [38,44,45] for child participants, by using a concept that was engaging to children, colorful stimuli, simplified instructions, and by reducing the duration of the experiment to 480 trials.

We recruited 13 children with CP aged 5–12 years old with Gross Motor Function Classification System (GMFCS) levels 1, 2 and 3 (6 females, 7 males; mean age = 7.77 years, SD = 2.05). Table 1 contains patient information. Two subjects with CP were excluded due to looking away from the screen. These participants did not complete the experiment. An additional two subjects with CP aged 5 years old were excluded because they had difficulty in using a computer mouse. These participants did not complete the experiment. We included an age-matched TD control for each patient with CP who completed the experiment. Therefore, the data of 18 participants were included in our final data set (CP group and TD controls after exclusion: 8 females, 10 males, mean age = 8.22 years, SD = 1.80). Participants with CP were recruited through a rehabilitation hospital and age-matched control data was from a previous study [46]. Participants were recruited from 12/01/16 to 03/01/17. All participants had self-reported normal or corrected vision.

**Table 1:** Patient information.

Patient	Age	Sex	GMFCS	MACS	Type	Etiology
1	5	F	2	2	Spastic hemiplegia (l ^a^)	Chromosomal deletion
2	5	M	2	2	Spastic hemiplegia (r)	Prematurity
3*	6	F	3	2	Spastic diplegia	Prematurity
4*	6	M	2	1	Spastic hemiplegia (l)	Polymicrogyra
5	7	F	2	1	Spastic diplegia	Prematurity
6*	7	F	1	1	Spastic diplegia	Prematurity
7*	8	M	2	1	Spastic diplegia	Prematurity
8*	8	M	3	2	Spastic quadraplegia	Prematurity
9*	9	F	1	1	Spastic diplegia	Prematurity
10*	9	M	2	2	Spastic hemiplegia (r)	Stroke
11*	9	M	2	2	Spastic hemiplegia (l)	Prematurity
12	10	F	2	2	Spastic diplegia	Prematurity
13*	12	M	2	3	Spastic diplegia	Prematurity

* patients included

^a^ For hemiplegic patients, *l* indicates that patients are more affected on the left side of the body and *r* indicates that they are more affected on the right side.

In a quiet room, participants sat in front of a 52 cm wide, 32.5 cm high computer monitor. Before starting the experiment, subjects were presented with the instructions that someone behind them was throwing “candy” (targets) into a “pond” (the screen). They were told that on each trial they should estimate where the candy target landed and that they should try to catch as many candy as possible over the course of the experiment. Candy targets were drawn from a Gaussian distribution centered at the middle of the screen, $Ν(\mu ,\sigma_{s}^{2})$, where *μ* is the mean of the prior distribution and $\sigma_{s}^{2}$ is its variance. On each trial, they were presented with an uncertain “splash” stimulus for one second and were told that the splash was caused by a hidden candy target. The splash was *n* = 4 samples from a Gaussian likelihood distribution that was centered on target location, $Ν(s,\sigma_{l}^{2})$, where *s* is the true target location and mean of the likelihood distribution and $\sigma_{l}^{2}$ is its variance. Subjects provided an estimate of the candy target’s location on the horizontal axis using a vertical bar that extended from the top to the bottom of the screen. We explained that the bar was a “net” that they could use to catch the candy target. The net appeared at the same time as the splash at a random location on screen. Subjects had 6 seconds to respond. After providing a response, they were shown the true candy location.

One simple strategy for performing sensorimotor estimation under uncertainty is to consistently judge target location at the center of the splash–i.e. full reliance on the likelihood. This strategy works well when the likelihood distribution is narrow, because the closely-spaced points of the splash are an accurate indicator of target location (Fig 1b, left). However, full reliance on the likelihood would cause a subject to miss targets more frequently as the likelihood distribution widens (Fig 1b, right). When sensory information is unreliable, rather than relying on the likelihood completely, we maximize performance by giving more weight to our prior belief on target location. More generally, the best or optimal strategy involves weighing sources of information according to their relative precision.

Formally, weighing sources of information according to their relative precision corresponds to Bayesian inference. An optimal Bayesian observer combines noisy sensory information from the likelihood, $Ν(s,\sigma_{l}^{2}/n)$ with a prior, $Ν(\mu ,\sigma_{s}^{2})$, resulting in a posterior distribution over target location, $N(\left(\frac{\mu }{\sigma_{s}^{2}}+\frac{s}{\sigma_{l}^{2}/n}\;\right)/(\frac{1}{\sigma_{s}^{2}}+\frac{1}{\sigma_{l}^{2}/n}),\;1/(\frac{1}{\sigma_{s}^{2}}+\frac{1}{\sigma_{l}^{2}/n}))$. The mean of the posterior is a mean of the prior and likelihood information, weighted by their precisions. From this posterior distribution, an estimate, $\hat{x}$, is computed. Therefore, the optimal reliance on the likelihood is a function of prior and likelihood uncertainties, $\sigma_{s}^{2}/(\sigma_{s}^{2}+\sigma_{l}^{2}/n)$. We can manipulate the prior and likelihood variances and measure their influence on subjects’ reliance on the likelihood, in order to investigate use of probabilistic information during sensorimotor estimation.

To investigate how children use probabilistic information during sensorimotor estimation, we manipulated the variances of prior distribution and likelihood distributions. We used a Gaussian prior distribution with a mean at the center of the screen and standard deviation of .03 (Narrow Prior) or .1 (Wide Prior) in units of screen width. The likelihood distribution was centered on target location and had a standard deviation of .025 (Narrow Likelihood), .1 (Medium Likelihood), or .25 (Wide Likelihood) in units of screen width. There were six conditions: Narrow Prior–Narrow Likelihood, Narrow Prior–Medium Likelihood, Narrow Prior–Wide Likelihood, Wide Prior–Narrow Likelihood, Wide Prior–Medium Likelihood, Wide Prior–Wide Likelihood.

The experiment consisted of four blocks, each lasting 120 trials. This was preceded by a practice block lasting 10 trials, which allowed participants to familiarize themselves with the task and practice using the computer mouse. Trials were blocked by prior condition, with all likelihood conditions being presented in randomized order within one block. The prior over target location switched from block to block with a randomly chosen starting condition for each participant (i.e., narrow-wide-narrow-wide or wide-narrow-wide-narrow).

We introduced a number of modifications to previous experiments [44,45] to engage child participants in the task. The visual stimuli were brightly colored. Participants were shown how much candy they had won on screen and participants won a "bonus" piece of candy for every ten candy they caught. Sounds were presented to signal successfully catching a target and missed responses when they did not respond within the 6-second time window. Step-by-step instructions were shown to participants on screen before the experiment, to ensure that all participants received the same instructions. We told participants that their payment or “prize” was based on the number of candy that they caught. The engaging task, the four short experimental blocks or ‘games’ each lasting 5–10 minutes, and the motivation in the form of a prize ensured that participants were not discouraged while doing the experiment.

Ethical approval was provided by the NU IRB #20142500001072 (Northwestern University, USA). A parent provided written informed consent for their child to take part and completed the Developmental Coordination Disorder questionnaire [47], a modified Vanderbilt questionnaire to assess for ADHD [48], and the Behavior Assessment System for Children, BASC-3, parent rating scales [49]. After the participant had completed the game, we administered the child mini-mental state evaluation [50].

### Data analysis

We were interested in the integration of probabilistic information from a prior distribution with sensory information during sensorimotor estimation. To investigate this, we examined whether samples from the likelihood distribution, *X* = {*x*_1_, *x*_2_, *x*_3_, *x*_4_}, were combined with information about the prior distribution, $Ν(\mu ,\sigma_{s}^{2})$, when producing an estimate of target location, *ŝ*. We quantified this for each condition using the extent to which participants relied on the likelihood, given by the linear relationship between the centroid of the splash, $c=\sum_{i}^{n}x_{i}/n$, and their estimate on each trial, *ŝ*. We performed a linear regression with estimates, *ŝ*, as the dependent variable and the likelihood centroid, *c*, as the independent variable, which resulted in a measure of reliance on the likelihood, which we term the *Estimation slope*. We assumed that subjects accurately learned the mean of the prior, and set the intercept to 0. If participants relied only on the likelihood to form their estimate, then, participants should point close to the centroid of the splash, *c*, on all trials, leading to an *Estimation slope* ≈ 1. If instead participants ignore the likelihood and instead only use their learned prior, then their estimates should not depend on the *c*, leading to an *Estimation slope* ≈ 0. Therefore, from participants’ estimates we obtain a measure of their reliance on the likelihood or prior.

The theoretical variance of the likelihood and prior used in the experiment provide optimal values for the *Estimation slope*, i.e. how much participants should rely on the likelihood. For an optimal Bayesian observer, sources of information are weighed according to their relative reliabilities, *Estimation slope* $opt\;=\sigma_{s}^{2}/(\sigma_{s}^{2}+\sigma_{l}^{2}/n).\;$ Optimal *Estimation slope* values allowed us to know what participants should do to maximize their performance under uncertainty.

Since the data did not meet the requirements for parametric tests, we used the non-parametric Scheirer-Ray-Hare test and non-parametric Wilcoxon signed rank tests for comparisons.

## Results

We wanted to investigate whether Bayesian integration is impaired in CP. To do so, we examined whether children with CP aged 6–12 years old and age-matched controls differed in how they integrated prior and likelihood information during sensorimotor estimation (Fig 1). We examined the use of probabilistic information by quantifying subjects’ task performance and subjects’ reliance on the likelihood using an *Estimation slope* measure.

**Fig 2:**
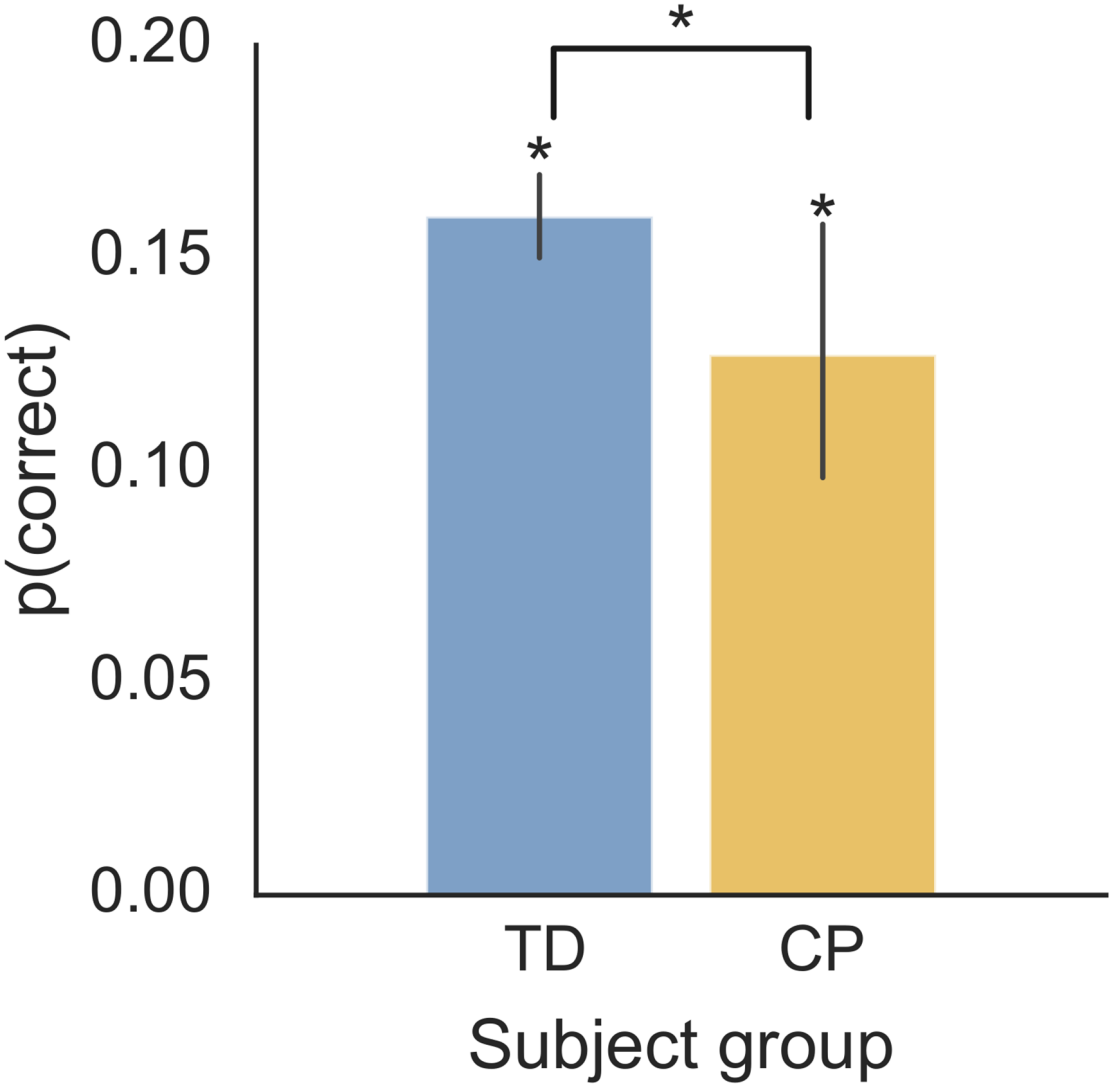
Performance of the candy-catching task. The mean proportion of correct responses, *p(correct)*, is shown as for controls and participants with CP, with error bars showing the 95% confidence intervals (CI).

We first examined performance on the candy-catching task. It was important to establish that subjects understood and carried out the task, with performance well above the chance level of 2%. We therefore compared the proportion of candy caught, *p(correct)*, to chance level in TD control and CP groups (Fig 2). The performance of both groups exceeded chance level, as tested by non-parametric Wilcoxon signed-rank tests with Bonferroni-corrected p-values (Controls: median = .16, W = 0, p = .01, CP: median = .12, W = 0, p = .01). This showed that all age groups understood and carried out the candy-catching task. Next, we asked if there is an effect of CP on performance (Fig 2). Performance, *p*(*correct*), is significantly lower in children with CP relative to controls (W = 19, p = .03). Therefore, task performance of both groups was above chance, with a significant difference between groups.

**Fig 3:**
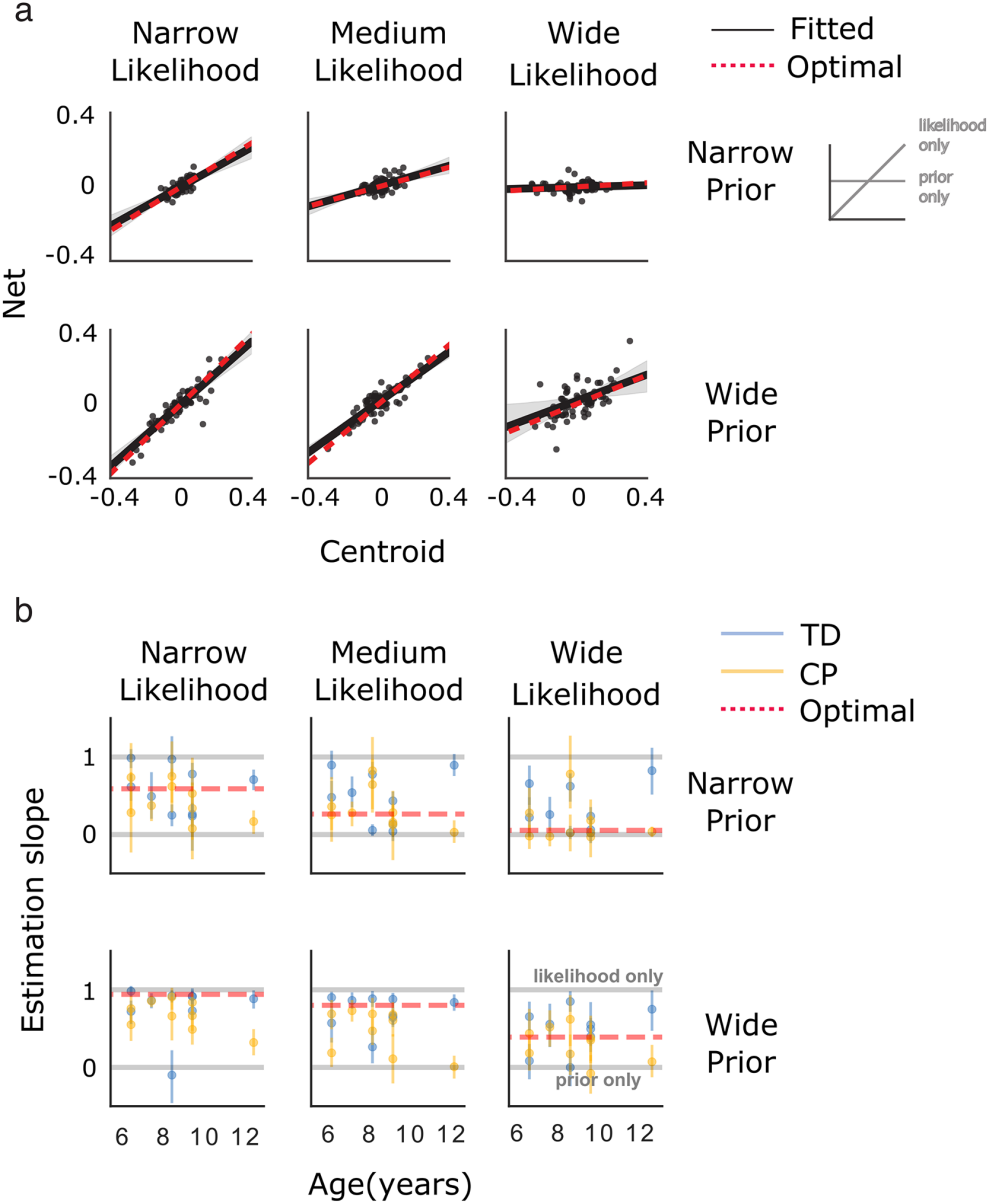
Estimation data. (**a**) Estimation data overlaid with linear fit for a representative subject (child with CP aged 9 years old). The net position as a function of the centroid of the likelihood is shown for each trial (points). The fitted *Estimation slope* (black line) and the optimal *Estimation slope* (dashed red line) are displayed. Each panel displays estimation data for one condition, as defined by prior and likelihood width. (**b**) The mean bootstrapped *Estimation slope* is shown for children with CP and TD controls (error bars = 95% CI). The optimal *Estimation slope* values are shown (dashed red line).

In order to investigate sensorimotor estimation under uncertainty, we manipulated the variance of the prior and likelihood, then measured the *Estimation slope* in each condition. In order to quantify the *Estimation slope*, we estimated the relationship between the centroid of the likelihood on each individual trial, *c*, and estimates, *ŝ*. Full reliance on the likelihood would indicate a close relationship between estimates and the likelihood, *Estimation slope* ≈ 1. Full reliance on the prior would indicate a lack of relationship between estimates and the likelihood, *Estimation slope* ≈ 0. To estimate the *Estimation slope* from the data, we performed linear regression on the data of individual subjects for each condition, as defined by the prior width and likelihood width. The fitting procedure provided reasonable fits to the data for individuals (shown for a 9-year old with CP, Fig 3a), and across the entire data set (Fig 3b). This allowed us to quantify the nature of integration of prior and likelihood for each subject.

**Fig 4:**
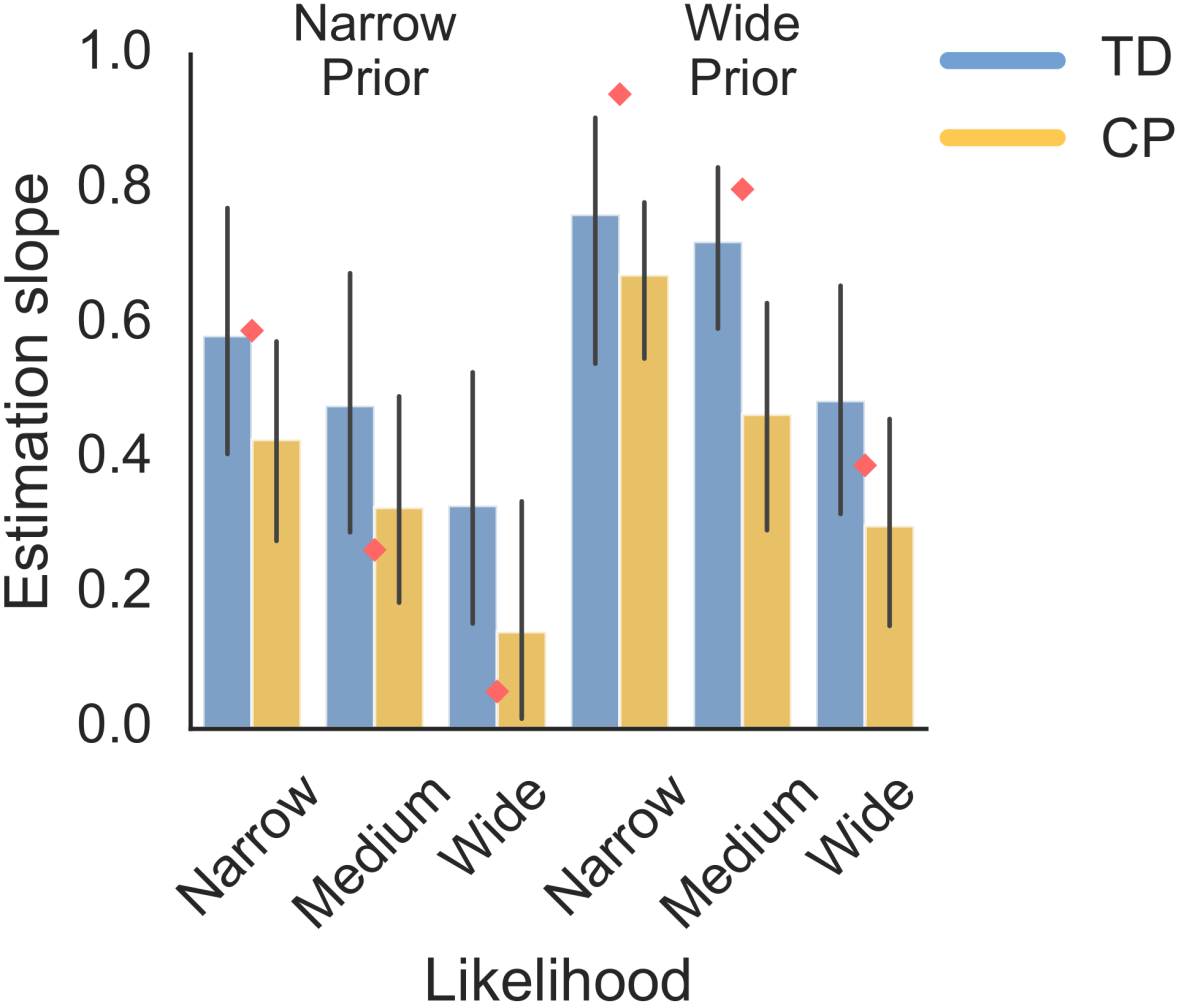
Estimation slopes. *Estimation slope* as a function of prior and likelihood for children with CP and age-matched TD controls. The mean *Estimation slope* is shown with error bars displaying the 95% CI. The optimal *Estimation slope* in each condition shown by red diamonds.

We wanted to examine the influence of prior and likelihood variance on sensorimotor estimation in children with CP (Fig 4). To do so, we examined the influence of prior variance, likelihood variance and subject group (CP or TD) on the *Estimation slope*. There was a significant main effect of prior variance (χ^2^(1) = 9.14, p = .003) and likelihood variance (χ^2^(2) = 17.30, p = .0002) on the *Estimation slope*. Therefore, subjects used the prior and likelihood in their estimates. There was also a significant main effect of subject group (χ^2^(1) = 8.30, p = .004). This showed that children with CP had an overall tendency to ignore likelihood information (Fig 4), which may account for the increased performance in controls. However, no interactions were significant, meaning that there is no evidence for a difference in use of prior and likelihood in CP. In sum, subjects used the prior and likelihood when producing estimates, but there was no difference found between CP and control subjects in their use of probabilistic information, except for an overall tendency for children with CP to ignore the likelihood.

We did not exclude experimental data on the basis of questionnaire and neuropsychological test data or provide analysis of how integration relates to motor ability using questionnaire data. The purpose of administering Vanderbilt questionnaire, BASC-3 and child mini-mental state evaluation was participant screening. However, we found that the participants who were inattentive during the experiment completed less than 50 trials of the experiment and were therefore excluded on this basis. The Developmental Coordination Disorder questionnaire was administered because we planned to examine whether differences in motor abilities within control and patient groups influenced the integration of probabilistic information. However, since we found no evidence for a difference between CP and TD control groups, and these groups show clear differences in motor abilities, we did not look for effects based on more subtle differences within groups.

## Discussion

We examined whether Bayesian integration during sensorimotor estimation is disrupted in CP. The performance of both children with CP and TD controls was above chance. Therefore, both groups understood and carried out the task. When we investigated use of probabilistic information by examining the *Estimation slope*, we found significant use of information from the prior and from the likelihood in producing estimates, and no evidence for a difference between CP children and TD controls, as shown by a lack of significant interaction between subject group and prior or likelihood conditions.

We found that integration of probabilistic information was the same in CP and TD children. While we may have failed to uncover subtle differences between groups in our experiment, generally speaking, children with CP used information from the prior and likelihood when performing sensorimotor estimation. That said, it is likely that there are experimentally testable conditions under which sensorimotor processing is different in children with CP. Due to a lack of successful motor-learning experiences during development, children with CP may find it more difficult to learn priors (in our task the prior was shown on screen), or to learn priors with more complex shapes. Children with CP could have different internal models of their own movement variability, with consequences for motor adaptation [51]. They could have difficulty with more complex motor tasks that involve planning [52,53], or in dynamic tasks, where subjects must make continual adjustments [54]. More research is needed to define the sensorimotor processes that are disrupted in children with CP. From our results we can conclude that Bayesian integration is not disrupted.

The only observable difference between children with CP and TD controls was a subtle tendency for children with CP to ignore likelihood information relative to TD controls. While this could indicate greater reliance on the prior in CP children, there are other factors which could contribute to this difference. The initial position of the net (vertical bar used to provide estimates) was randomized. This result could simply mean that there were more trials where children with CP did not move the net from its initial position, or more trials where the child moved the net to a random location on screen. This could be tested using trials where the initial position of the net is at the likelihood’s center. If there is evidence for children with CP adjusting more toward the prior under these task conditions than controls, it may genuinely be the case that children with CP have a greater reliance on the prior.

The lack of evidence for a difference in use of statistical information between children with CP and TD children does not imply full use of statistical information by either subject group. As we have shown in a previous study [46], use of probabilistic information shifts toward the statistical optimum over the course of development. Our previous findings showed that while use of likelihood information is present in young children aged 6–8 years old, the use of the prior distribution changes over the course of development, reaching adult-like performance at 9–11 years [46]. Therefore, the results of this study do not imply that the children perform sensorimotor estimation in a fully statistically-efficient way, but that children with CP do not deviate further from the statistical optimum than TD children.

In the present study, we addressed sensorimotor integration in a small sample of children with CP. CP is a heterogeneous disorder. Therefore, our small sample size limits our ability to generalize findings to patients belonging to all CP subtypes and degrees of severity. In future investigations, it could be interesting to consider the relationship between specific brain injuries during development and sensorimotor processing. While injury is variable across cases of CP and brain injury is often diffuse, dystonic CP is known to involve a specific basal ganglia component [55], and may have a stronger effect on sensorimotor integration. Further investigation into the relationship between sensorimotor processing and injury type [56], or the strength of specific symptoms in a larger, more diverse sample of patients may be informative on how specific sensorimotor deficits play a causal role in generating symptoms of motor dysfunction.

Our findings have general implications for the development of clinical interventions. Therapeutic interventions should use unimpaired skills in order to augment motor function, allowing for a small set of functions to be leveraged into a more significant set of skills [57]. We found that children with CP use statistical information in the same manner as age-matched controls. This is supportive of interventions which exploit sensory integration by providing patients with sensory cues to improve the timing and accuracy of movement [42,43,58]. Since children with and without CP are comparable in their treatment of statistical information, this also suggests that some strategies which improve motor learning in TD children may generalize well to children with CP.

We have shown that Bayesian integration of prior and likelihood information is not disrupted in CP. This is consistent with the previous finding that children with CP can integrate sensory stimulation with information on joint position [42,43], which suggests that children with CP can bring together different sources of information to control movement. Here, our contribution was to examine the use of probabilistic information explicitly by examining sensorimotor estimation under conditions of experimentally-controlled uncertainty. We conclude that CP does not lead to impaired Bayesian integration. This also implies that brain injury in CP, which produces altered sensory activation and abnormal connectivity [16–20], does not affect processes which are needed for the representation of uncertainty in the brain [44].

## Supporting information

S1 File Patient information. (CSV)

S2 File Estimation data. (CSV)
